# Enhancement of Interferon-γ Secretion by *Lepidium meyenii* Extract Supplementation After Exhaustive Endurance Exercise in Healthy Men: A Double-blind, Placebo-controlled Trial

**DOI:** 10.7150/ijms.104812

**Published:** 2025-01-01

**Authors:** Pei-Wei Weng, Yu-Chun Chung, Tso-Ching Lin, Pei-Chia Hsu, Cheng-Tse Yang, Shan Lin, Ming-Ta Yang

**Affiliations:** 1 Department of Orthopaedics, School of Medicine, Taipei Medical University, Taipei 110301, Taiwan.; 2 International PhD Program in Biomedical Engineering, Taipei Medical University, Taipei 110301, Taiwan.; 3 Center for General Education, Taipei Medical University, Taipei 110301, Taiwan.; 4 Department of Sport and Health Management, Da-Yeh University, Changhua 515006, Taiwan.; 5 School of Public Health, Taipei Medical University, Taipei 110301, Taiwan.; 6 The OSU Interdisciplinary PhD Program in Nutrition, The Ohio State University, Columbus, OH 43210, USA.; 7 Graduate Institute of Medical Sciences, Taipei Medical University, Taipei 110301, Taiwan.; 8 Clinical Research Center, Taipei Medical University Hospital, Taipei 110301, Taiwan.

**Keywords:** Lymphocyte, Interferon-γ, Peripheral blood mononuclear cells, CD4^+^/CD8^+^ ratio

## Abstract

**Aim:** To investigate the effects of 12-week *Lepidium Meyenii* extract supplementation on immune responses and inflammatory cytokines after exhaustive endurance exercise (EEE), emphasizing its novel focus on peripheral blood mononuclear cells (PBMCs) cytokine secretion and the implications of interferon-γ (IFN-γ) as a marker for immune modulation.

**Methods:** Twenty healthy men were recruited and assigned into maca and placebo groups using a matched-pair design based on their maximal oxygen consumption (V̇O_2max_). All participants consumed 2.25 g of maca or placebo twice per day for 12 weeks, and they then performed EEE. Researchers collected blood samples before exercise, immediately after exercise, and at 2, 4, and 24 hours post-exercise to analyze immune functions and inflammatory markers.

**Results:** No significant differences were observed in the variables between the two groups before supplementation. However, interferon-γ levels from peripheral blood mononuclear cells were significantly higher in the maca group than in the placebo group immediately and at 24 hours after exercise. Regarding the main time effect, the number of lymphocytes in all participants was significantly lower at 2 and 4 hours after exercise than before supplementation. The CD4^+^/CD8^+^ ratio in the groups was significantly lower immediately after exercise than before supplementation, and the ratio retuned to baseline levels at 2 hours after exercise.

**Conclusion:** A 60-minute EEE session induces the open window phenomenon, characterized by immune suppression. Moreover, 12-week maca supplementation had positive effects only on interferon-γ levels from peripheral blood mononuclear cells.

## Introduction

During prolonged intense exercise, all white blood cell subgroups increase. However, within 1-2 hours of recovery after EEE, lymphocyte counts drop below pre-exercise levels 1-2. This reduction denotes immune suppression, which affects lymphocyte subgroups. Lymphocytes comprise various subgroups, including T cells, B cells (CD19^+^), and natural killer cells (CD56^+^). T cells are divided into helper T cells (CD4^+^) and cytotoxic T cells (CD8^+^). CD4^+^ T cells play a crucial role in immune regulation, and they differentiate into Th1 and Th2, which participate in cell-mediated immunity and humoral immunity, respectively 3. Th1 secretes cytokines such as interferon-gamma (IFN-γ) and interleukin-2 (IL-2) for regulating immune responses 4. Conversely, Th2 releases IL-4 for regulating humoral immunity and increasing the secretion of anti-inflammatory cytokines. IL-4 reduces the expression of Fc receptors on the cell surface, thereby diminishing antibody-dependent cellular cytotoxicity and macrophage functions. IL-4, in combination with IFN-γ, often exerts antagonistic effects 5. CD8^+^ T cells exhibit cytotoxicity, attacking damaged or virus-infected cells. Intense exercise has been demonstrated to reduce the number and activity of CD56^+^ cells 6, and it affects the quantity of Th1 cells and their secretion of IFN-γ and IL-2. In addition, the secretion of the anti-inflammatory cytokines IL-4 and IL-10 increases after exercise 3. During intense exercise, the balance of cytokines secreted by immune cells shifts, resulting in a decrease in the Th1/Th2 ratio and indirectly suppressing the proliferation of CD19^+^ cells 3,7. During intense exercise, individuals may be more susceptible to pathogenic infections, a concept known as the "open window phenomenon," which describes transient immune suppression following exhaustive physical activity 8, which indicates that exercise potentially affects the immune system.

The secretion of cellular hormones such as IL-6 during exercise plays a pivotal role, with the dual function of regulating the secretion of IL-10 and IL-1 receptor antagonists 9. That is, these hormones promote both proinflammatory and anti-inflammatory responses, leading to processes such as vascular regeneration and myonuclear proliferation and influencing processes such as fat break-down, insulin sensitivity, and glucose uptake 10. However, athletes engaged in prolonged high-intensity endurance exercise may experience microtrauma resulting in microinjuries to the connective tissues, bones, and skeletal muscles 11. Excessive inflammatory reactions have adverse effects, diminishing subsequent exercise performance and overall quality of life. Studies on marathon races of different distances have found variations in the changes in cytokines within 24 hours postexercise. After marathon races, levels of IL-6, IL-8, TNF-α, and IL-10 increased; levels of IL-2 and IFN-γ decreased; and no significant change was detected in IL-4 levels 12. These findings suggest a sustained impact of vigorous endurance exercise on cytokine levels, although no consensus has been reached regarding such an impact. Additionally, research indicates that appropriate nutrient supplementation postexercise may aid in precisely regulating the immune system, mitigating inflammatory responses and consequently enhancing exercise performance 13. Therefore, the International Olympic Committee has included nutritional supplementation in its prevention and response guidelines, indicating it is imperative for managing physiological stress and preventing disease onset.

Through continuous progress in academic research, scientists have discovered benefits of maca beyond those it is traditionally used to achieve; these benefits include antifatigue, antioxidant, anti-inflammatory, neuroprotective, and memory improvement effects 14. Additionally, maca was reported to exhibit potent immune-regulating and hepatoprotective activities 15. The main active components identified in maca include glucosinolates, thiohydantoins, macenes, macamides, polysaccharides, and alkaloids 16. In the past decade, maca has emerged as a highly promising health-promoting ingredient. In the literature on the immune-modulating function of maca, studies have mainly focused on maca polysaccharides targeting macrophages for regulating immune responses; research on maca polysaccharides targeting other immune cells is scarce. In cellular experiments conducted by Zhang *et al.* (2017), maca induced polarization of initial macrophages into M1 macrophages, which phagocytized pathogens and reduced inflammation 17. Chen *et al.* (2022) conducted in vitro experiments on intestinal epithelial cells, and they found that maca enhanced immune function by promoting the secretion of IL-8, IL-10, and IFN-γ by intestinal epithelial cells 18. This result indicates the potential applications of maca for individuals with diminished immune capacity or those undergoing tumor treatment. Furthermore, through mouse experiments, Fei *et al.* (2022) discovered that maca delayed declines in white blood cell counts, reduced the inhibition rates of lymphocytes in the spleen, and modulated immune responses by altering the cytokine balance 19. Regarding the application of maca in research on exercise performance, human studies are scarce, with the majority of studies being conducted using animals and cells. Lee *et al.* (2023) found that 8 weeks of supplementation with black maca extract exerted beneficial effects on biochemical indicators and exercise performance in elite athletes 20. Other studies have found that maca supplementation resulted in varying degrees of reduction in serum inflammatory response markers in various forms of exercise, including fin swimming and racket sports. Additionally, maca supplementation has been reported to exert positive effects on muscle fatigue, muscle endurance, and muscle strength 21-23. Current research on maca predominantly focuses on the changes in immune function and inflammatory responses, particularly in terms of macrophage regulation and neuroinflammatory responses, induced by maca. Additional human studies should be conducted to clarify the specific changes in immune function and inflammatory responses following EEE. Therefore, we hypothesized that maca extract supplementation would have positive effects on immune responses and inflammatory cytokines following EEE.

## Materials and Methods

### Participant selection criteria

This study enrolled 24 healthy men. Individuals with a history of cardiovascular diseases, liver diseases, kidney diseases, diabetes, or immune-related diseases as well as those with a history of acute exercise injuries were excluded. Participants were stratified into maca and placebo groups by using a mixed-design approach. Before the experiment, the participants were provided with information on the study protocol, and they were asked to complete a health questionnaire and sign an informed consent form. The inclusion criterion was not having participated in any experiment or research project in the 3 months prior to the start of the present study. Throughout the experimental period, participants were prohibited from using any form of nutritional supplement, and their dietary and physical activity patterns were maintained as a part of their daily routine; they were also required to abstain from alcohol consumption. The final analysis included 20 participants, with 9 in the maca group and 11 in the placebo group. The data of participants who withdrew from the study were excluded from the analysis. No significant differences were observed in the basic characteristics, including age, body height, body weight, physical fitness levels (e.g., V̇O_2max_), and exercise intensity between the two groups (**Table 1**). These findings suggest that the groups were well-matched at baseline, minimizing potential confounding effects on the outcomes. Additionally, the study was registered on ClinicalTrials.gov (ID number: NCT05779410).

### Experimental design and procedure

**Figure 1:**
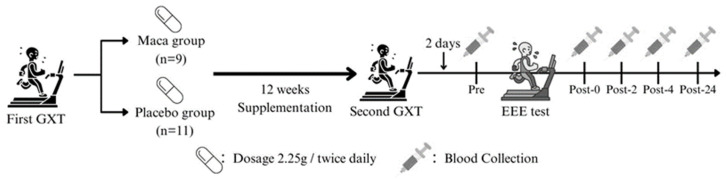
Experimental scheme. GXT, graded exercise test; EEE, exhaustive endurance exercise; Pre, before EEE test; Post-0, immediately after EEE test; Post-2, 2 hours after EEE test; Post-4, 4 hours after EEE test; Post-24, 24 hours after EEE test.

Participants underwent an initial graded exercise test (GXT) on a treadmill to determine their V̇O_2max_. Based on their V̇O_2max_ results, participants were assigned to either the maca or placebo group using a matched-pair design. This approach ensured equivalence in baseline aerobic capacity and fitness levels between the two groups, thus controlling for potential confounding effects related to individual differences in fitness. While this design did not involve random assignment, it was chosen to ensure methodological rigor and reliability in evaluating the specific effects of maca supplementation on immune and cytokine responses. All participants underwent a second GXT to collect V̇O_2max_ data, which served as a reference for determining the individualized exercise intensity of EEE after 12-week supplementation. All participants performed EEE test 2 days after the second GXT, following a mandatory 8-hour fasting period and consuming a standardized meal (130 g of bread accompanied by 450 mL of rice and peanut milk, providing approximately 648 kcal) 1 hour prior to the test. Blood samples were collected from the antecubital vein at the following time points: before EEE (Pre), immediately postexercise (Post-0), 2 hours postexercise (Post-2), 4 hours postexercise (Post-4), and 24 hours postexercise (Post-24). **Fig. 1** illustrates the scheme of the study.

### Supplementation

Throughout the 12-week supplementation phase of this experiment, participants in the maca group consumed 2.25 g of maca extract capsules (Panion & BF Biotech Inc., Taipei, Taiwan) twice per day, after breakfast and after dinner, with a total dosage of 4.5 g/day. The maca extract utilized in this study was derived exclusively from the root of maca, sourced from the Junín region in Peru. The supplementation was produced as a light brown powder through processes including selection, cleaning, organic disinfection, crushing, extrusion, drying, and milling. The placebo group ingested corn starch capsules (Panion & BF Biotech Inc., Taipei, Taiwan) that were visually indistinguishable from the maca extract capsules.

### GXT protocol

The Life Fitness-Integrity CLST Treadmill was employed for comprehensive exercise testing. The participants initially performed a 5-minute warm-up exercise on the treadmill at a speed of 7.0 km/h, with this followed by a strategically timed 3-minute rest. In the subsequent running phase, the participants initially performed exercise at a speed of at 7.2 km/h, with incremental increases of 1.8 km/h every 2 minutes until the point of exhaustion. The oxygen consumption (V̇O_2_), carbon dioxide elimination (V̇CO_2_), and exchange rates of the participants were measured using a portable gas analyzer (MetaMax 3B; Cortex Biophysik, Leipzig, Germany). Additionally, their heart rates were recorded using a heart rate monitor (Polar S610, Kempele, Finland). In each stage, the participants rated their perceived exertion on the standard 6-20 Borg scale. Volitional exhaustion was determined when at least two of the following three criteria were met: (1) the rating of perceived exertion was ≥18; (2) the maximum heart rate was maintained within 15 beats/min of the predicted value; and (3) the respiratory exchange ratio was >1.1 24.

### Exhaustive endurance exercise test

On the testing day, the participants were provided with a standard breakfast and lunch at the laboratory. The participants rested for 30 minutes after breakfast (before pre-exercise blood collection). Subsequently, the participants engaged in a 5-minute warm-up on the treadmill at a speed of 7.0 km/h, with this followed by a 60-minute endurance exercise test at 70% of V̇O_2max_. At the conclusion of the 60-minute running test, the participants were asked to increase the intensity to 90% of V̇O_2max_ until they reached exhaustion.

### Biochemical variables

For immune cell analysis, a total sample of 9 mL of venous blood was collected into EDTA-containing tubes at Pre, Post-0, Post-2, Post-4, and Post-24. Analysis of white blood cells, neutrophils, lymphocytes, CD4^+^, CD8^+^, CD19^+^, and CD56^+^ was conducted following the methodology established in our previous research 25. For an analysis of cytokines secreted by PBMCs, blood samples were processed under aseptic conditions, and isolation and culturing of PBMCs were performed following a modified protocol described by Viora *et al.* 26. The supernatants were collected after 24 hours. The levels of TNF-α, IFN-γ, IL-2, IL-4, IL-6, IL-8, and IL-10 were measured using a BioLegend kit (ELISA MAX™Deluxe Set Human, BioLegend, San Diego, CA, US) following the manufacturer's instructions.

### Statistical analysis

Data processing and analysis were conducted using IBM SPSS version 22.0. Data are expressed as means ± standard deviations (means ± SDs). An independent-sample *t* test was used to compare the differences in participant characteristics, secondary GXT data (at baseline and 2 days before the end of supplementation), and the intensity of EEE between the groups. A 2 (groups: maca and placebo) × 5 (time points) two-way, mixed-design analysis of variance was used to compare the differences in immune function and inflammatory response variables between the two groups. When a significant interaction was noted, a simple test of the main effect was performed. If the interaction was nonsignificant, tests of the main effect were conducted. The significance level was set at α = 0.05.

## Results

### Effects of maca extract supplementation on immune responses following EEE

#### Changes in white blood cell subsets following EEE

**Figure 2:**
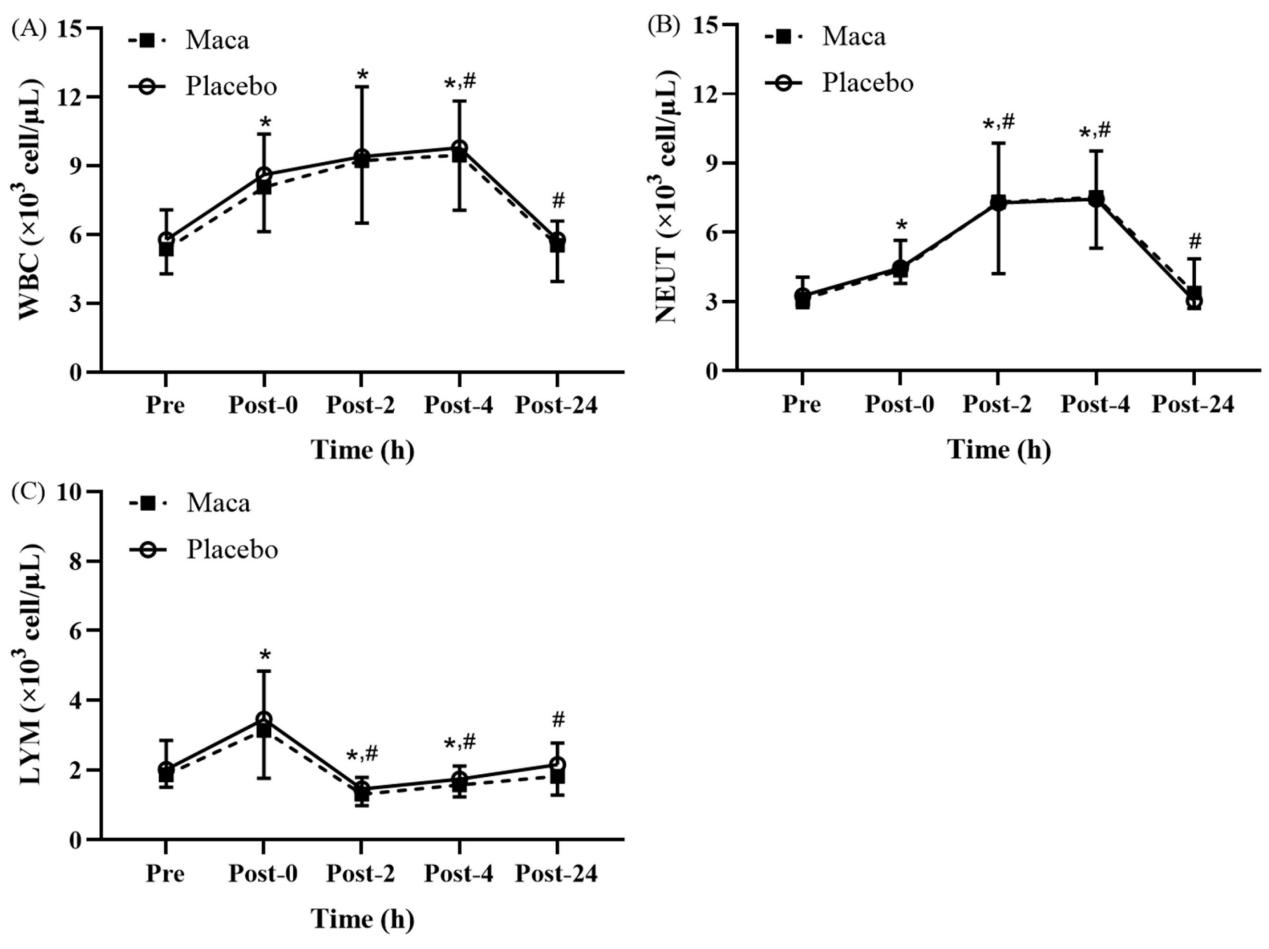
Exercise-Induced Changes in (A) white blood cell (WBC), (B) neutrophil (NEUT), and (C) lymphocyte (LYM) counts. Counts increased significantly at Post-0 and returned to baseline by Post-24. *p < 0.05 compared with baseline. #*p* < 0.05 compared with Post-0. Abbreviations: WBC, white blood cell; NEUT, neutrophil; LYM, lymphocyte; Pre, baseline (before exhaustive endurance exercise); Post-0, immediately after exhaustive endurance exercise; Post-2, 2 hours after exhaustive endurance exercise; Post-4, 4 hours after exhaustive endurance exercise; Post-24, 24 hours after exhaustive endurance exercise.

Significant time main effects were observed for the white blood cell, neutrophil, and lymphocyte counts at various time points before and after EEE (**Fig. 2A-2C**). No significant difference was observed between the groups, and no significant interaction was detected between groups and time. As presented in **Fig. 2A**, the white blood cell counts for all participants were significantly higher at Post-0, Post-2, and Post-4 than at baseline (*p* < 0.05). Additionally, the counts were significantly higher at Post-4 than at Post-0 (*p* < 0.05), and the counts returned to the baseline level at Post-24. As illustrated in **Fig. 2B**, the neutrophil counts for all participants were significantly higher at Post-0, Post-2, and Post-4 than at baseline (*p* < 0.05). The white blood cell counts were also significantly higher at Post-2 and Post-4 than at Post-0 (*p* < 0.05), and they returned to the baseline level at Post-24. As depicted in **Fig. 2C**, the lymphocyte counts for all participants were significantly higher at Post-0 than at baseline (*p* < 0.05), and the lymphocyte counts significantly decreased at Post-2 and Post-4 compared with the baseline (*p* < 0.05) and Post-0 (*p* < 0.05) counts. Moreover, the counts returned to baseline levels at Post-24. These results indicate that the EEE designed in this study induced immune suppression, consistent with the open window phenomenon, and triggered an inflammatory response within 24 hours.

#### Changes in lymphocyte subsets following EEE

**Figure 3:**
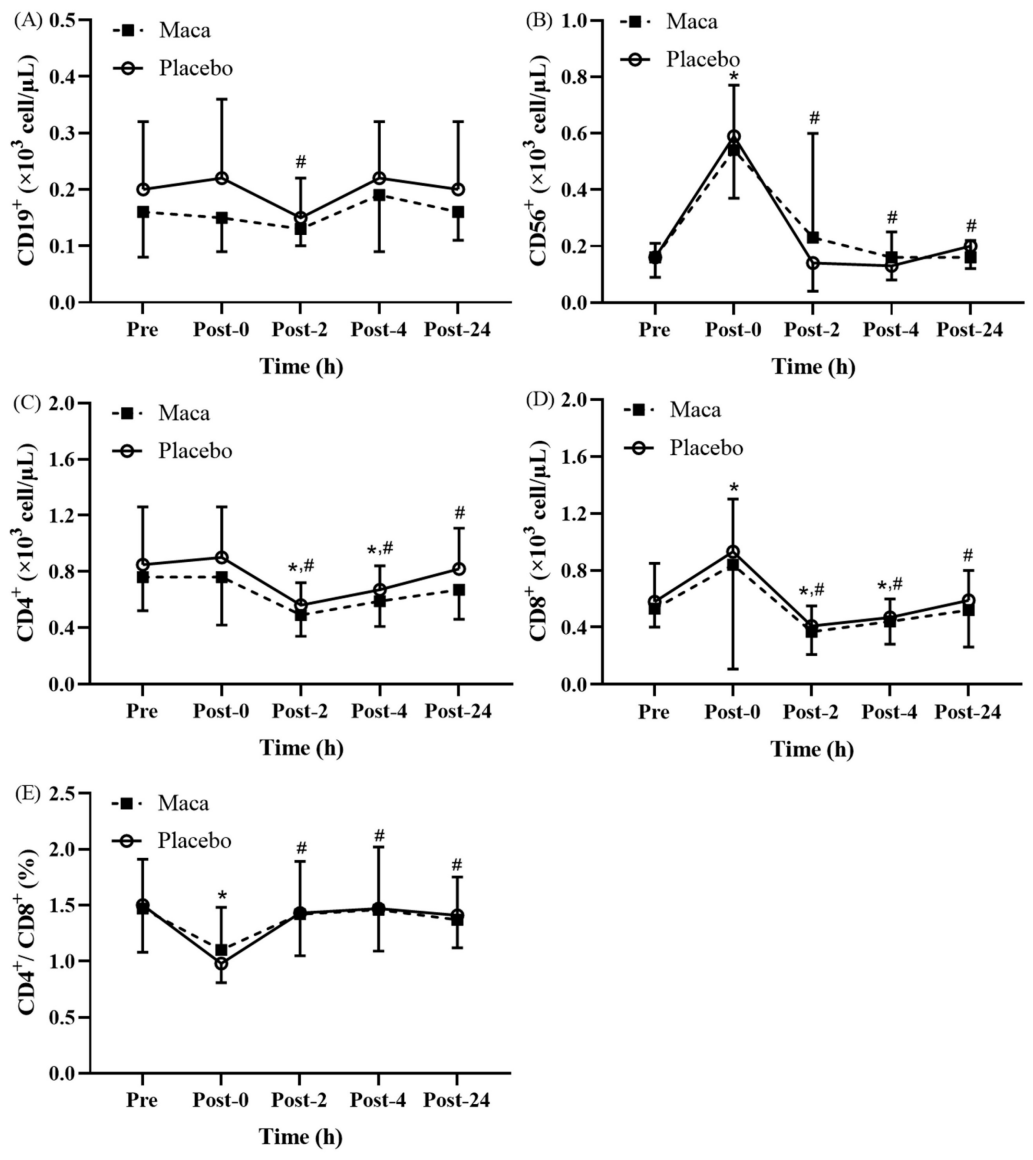
Exercise-Induced Changes in (A) CD19^+^ counts, (B) CD56^+^ counts, (C) CD4^+^ counts, (D) CD8^+^ counts, and (E) the CD4^+^/CD8^+^ ratio. Counts of CD19^+^, CD56^+^, CD4^+^, and CD8^+^ cells fluctuated significantly at Post-0, showing recovery trends at later time points. *p < 0.05 compared with baseline. #*p* < 0.05 compared with Post-0. Abbreviations: Pre, baseline (before exhaustive endurance exercise); Post-0, immediately after exhaustive endurance exercise; Post-2, 2 hours after exhaustive endurance exercise; Post-4, 4 hours after exhaustive endurance exercise; Post-24, 24 hours after exhaustive endurance exercise.

The results revealed significant time main effects on the counts of individual lymphocyte subsets at various time points before and after EEE (**Fig. 3A-3E**). No significant difference was observed between the groups, and no significant interaction was detected between groups and time. As depicted in **Fig. 3A**, the CD19^+^ counts for all participants were significantly lower at Post-2 than at Post-0 (*p* < 0.05), and nonsignificant differences were observed across the other time points. The CD56^+^ counts for all participants significantly increased at Post-0 compared with the baseline counts (*p* < 0.05), and the counts then significantly decreased at Post-2, Post-4, and Post-24 compared with the Post-0 counts (*p* < 0.05, **Fig. 3B**). As presented in **Fig. 3C**, the CD4^+^ counts for all participants were significantly lower at Post-2 and Post-4 than at baseline (*p* < 0.05), and the counts were significantly lower at Post-2, Post-4, and Post-24 than at Post-0 (*p* < 0.05). Subsequently, the counts returned to baseline levels at Post-24. The CD8^+^ counts for all participants were significantly higher at Post-0 than at baseline (*p* < 0.05), and the counts then significantly decreased at Post-2 and Post-4 compared with the baseline counts (*p* < 0.05, **Fig. 3D**). Moreover, the counts significantly decreased at Post-2, Post-4, and Post-24 compared with the Post-0 counts (*p* < 0.05), and at Post-24, the counts returned to those at baseline. As illustrated in **Fig. 3E**, the CD4^+^/CD8^+^ ratio for all participants was significantly lower at Post-0 than at baseline (*p* < 0.05). The ratio subsequently returned to that at baseline, and it became significantly higher at Post-2, Post-4, and Post-24 than at Post-0 (*p* < 0.05). Taken together, these results indicate that supplementation with maca extract does not significantly influence changes in the counts of lymphocyte subsets after EEE.

#### Changes in Th1/Th2 cytokine levels following EEE

**Figure 4:**
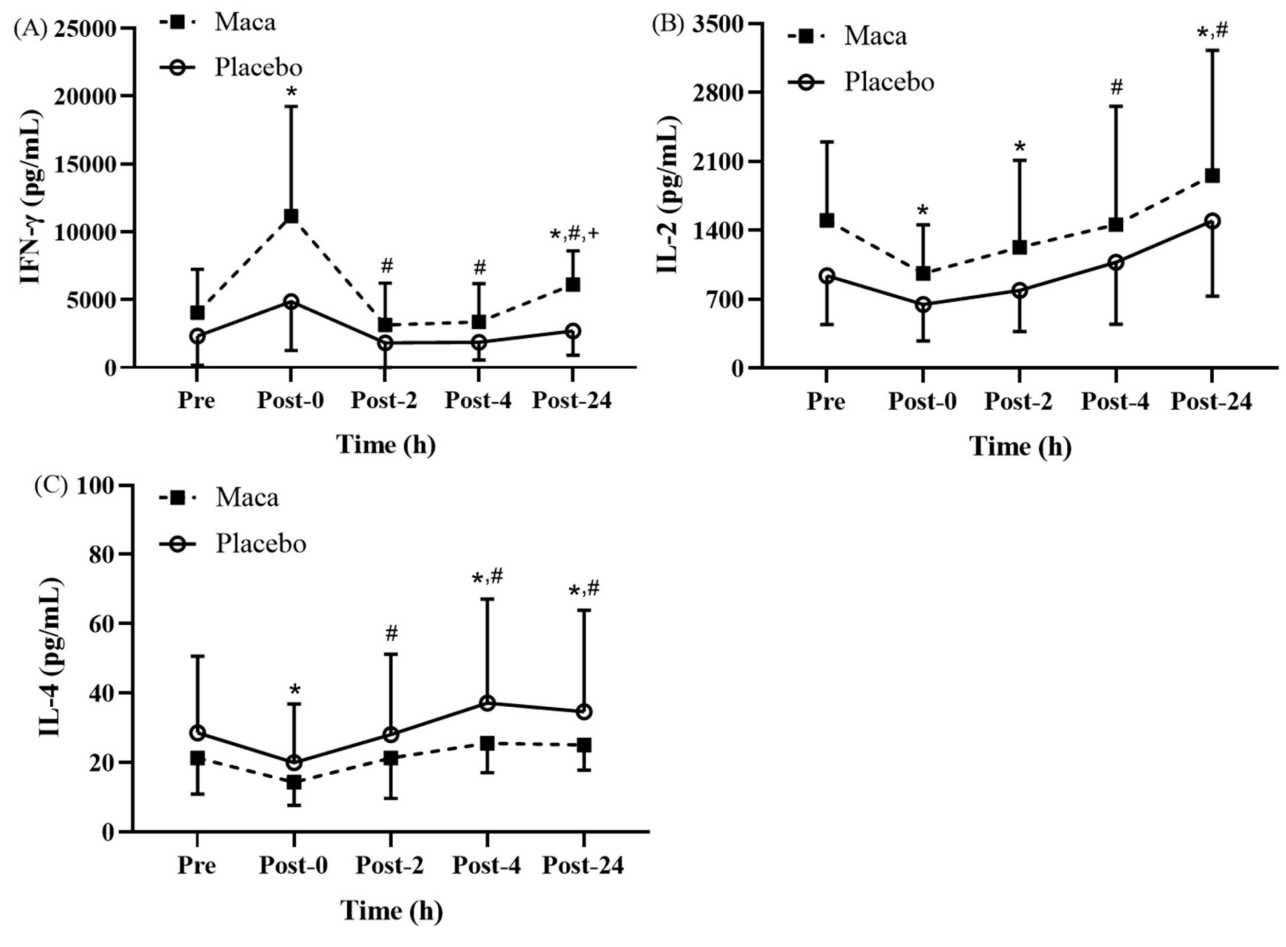
Exercise-Induced Changes in (A) IFN-γ, (B) IL-2, and (C) IL-4 levels. Th1 cytokine IFN-γ increased significantly at Post-0, while Th2 cytokine IL-4 remained unchanged. IL-2 showed a transient increase at Post-2. *p < 0.05 compared with baseline. #*p* < 0.05 compared with Post-0. +*p* < 0.05 compared with placebo. Abbreviations: IFN-γ, interferon-γ; IL-2, interleukin-2; IL-4, interleukin-4. Pre, baseline (before exhaustive endurance exercise); Post-0, immediately after exhaustive endurance exercise; Post-2, 2 hours after exhaustive endurance exercise; Post-4, 4 hours after exhaustive endurance exercise; Post-24, 24 hours after exhaustive endurance exercise.

This study identified significant time main effects on variations in the Th1/Th2 balance, which is represented by cytokine levels, at various time points before and after EEE (**Fig. 4A-4C**). As presented in **Fig. 4A**, for all participants, the time main effect on IFN-γ levels was significantly higher at Post-0 than at baseline (*p* < 0.05). Additionally, for both groups, the IFN-γ levels at Post-2, Post-4, and Post-24 returned to baseline levels, and these levels were significantly lower than the Post-0 levels (*p* < 0.05). Furthermore, the maca group exhibited significantly higher levels than the placebo group did at Post-24 (*p* < 0.05), and the differences between the two groups at Post-0 were nearly significant (*p* = 0.055). For all participants, the main time effect indicated that the IL-2 levels at Post-0 and Post-2 were significantly lower than the baseline levels (*p* < 0.05), and the levels were then significantly increased at Post-24 compared with the baseline and Post-0 levels (*p* < 0.05) (**Fig. 4B**). The main time effect indicated that for both groups, the IL-4 levels at Post-0 were significantly lower than the baseline levels (*p* < 0.05). In addition, relative to the baseline levels, the IL-2 levels increased at Post-4 and Post-24 (*p* < 0.05) (**Fig. 4C**), and the levels were significantly higher at Post-2, Post-4, and Post-24 than at Post-0 (*p* < 0.05). Thus, this study confirmed that supplementation with maca extract for 12 weeks could enhance the secretion of IFN-γ by PBMCs at Post-0 and Post-24. These findings suggest a potential positive effect on immune function. However, no significant changes were observed in IL-2 and IL-4 levels before and after exercise.

### Maca extract supplementation: impact on inflammatory response following EEE

#### Changes in proinflammatory cytokine levels following EEE

**Figure 5:**
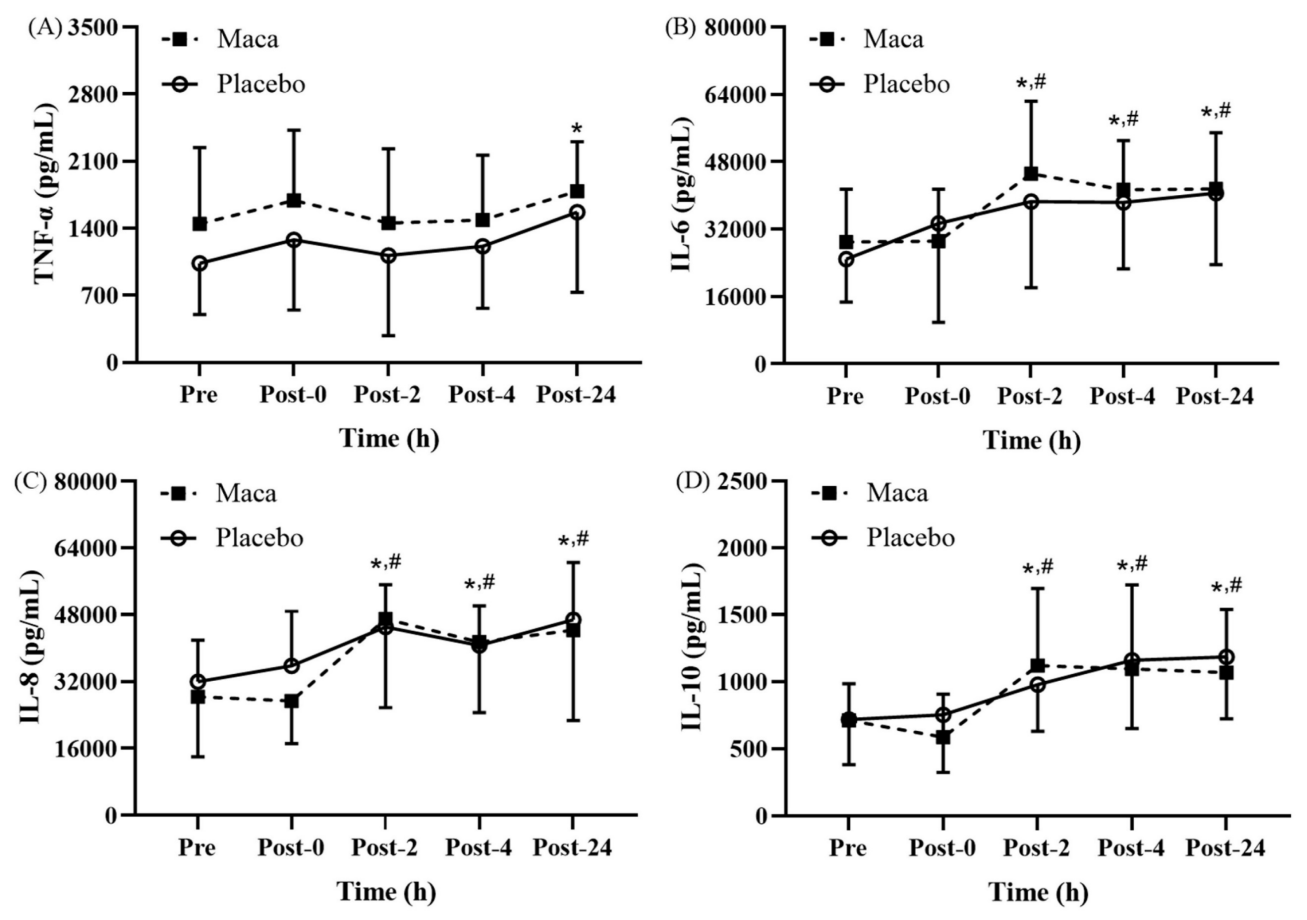
Exercise-Induced Changes in (A) TNF-α, (B) IL-6, (C) IL-8, and (D) IL-10 levels. Proinflammatory cytokines (TNF-α, IL-6, IL-8) significantly increased at Post-0 and returned to baseline by Post-24, while anti-inflammatory IL-10 levels remained unchanged. *p < 0.05 compared with baseline. #*p* < 0.05 compared with Post-0. Abbreviations: TNF-α, tumor necrosis factor-α; IL-6, interleukin-6; IL-8, interleukin-8; IL-10, interleukin-10. Pre, baseline (before exhaustive endurance exercise); Post-0, immediately after exhaustive endurance exercise; Post-2, 2 hours after exhaustive endurance exercise; Post-4, 4 hours after exhaustive endurance exercise; Post-24, 24 hours after exhaustive endurance exercise.

Significant time main effects on proinflammatory cytokine levels were observed at various time points before and after EEE (**Fig. 5A-5D**). As presented in **Fig. 5A**, for all participants, the TNF-α levels were only significantly higher at Post-24 relative to those at baseline (*p* < 0.05); no significant differences were observed at the other time points relative to at baseline or Post-0. As depicted in **Fig. 5B**, the IL-6 levels of all participants at Post-2, Post-4, and Post-24 were significantly higher than the baseline levels (*p* < 0.05), and the levels were also significantly higher than the Post-0 levels (*p* < 0.05). As illustrated in **Fig. 5C**, for both groups, the IL-8 levels were significantly higher at Post-2, Post-4, and Post-24 than at baseline (*p* < 0.05), and these levels were also significantly higher than the Post-0 levels (*p* < 0.05).

#### Changes in anti-inflammatory cytokine levels After EEE

As presented in **Fig. 5D**, for all participants, the IL-10 levels were significantly higher at Post-2, Post-4, and Post-24 than at baseline (*p* < 0.05), and the levels were also significantly higher than the Post-0 levels (*p* < 0.05). These results indicate that supplementation with maca extract did not exert beneficial effects on changes in the TNF-α, IL-6, IL-8, and IL-10 levels before and after exercise. This suggests that 12-week supplementation with maca extract might not influence the inflammatory response following EEE by modulating the levels of cytokines secreted by PBMCs.

## Discussion

This study investigated the effects of 12-week supplementation with maca extract on immune responses and inflammatory cytokines after EEE. The findings revealed that supplementation with 2.25 g of maca extract twice per day for 12 weeks solely exerted positive effects on IFN-γ secretion by PBMCs; such supplementation had no effect on other immune responses or cytokine indicators. A previous meta-analysis indicated that a single exercise session for >1 hour could suppress lymphocyte proliferation 27. However, other studies have revealed that the open window phenomenon of the immune response was induced by exercise with durations set at >2 hours 28-30. To the best of our knowledge, this is the first study indicating that a 60-minute EEE session can induce the open window phenomenon. Furthermore, our results indicate that for all participants, the white blood cell and neutrophil counts significantly increased at Post-0 and returned to baseline counts at Post-24 following EEE, which is consistent with the findings of previous research 31,32. Exercise increases cardiac output and blood flow, which alters hemodynamic shear stress and disrupts leukocyte-endothelial interactions. Catecholamines and glucocorticoids mobilize immune cells from storage areas, such as the vasculature, lungs, liver, and spleen, into peripheral circulation. This process leads to higher white blood cell and neutrophil counts 33,34. The current study discovered that for all participants, lymphocyte counts increased at Post-0 and decreased significantly below baseline levels at Post-2, aligning with the typical timing of immune suppression associated with the open window phenomenon as described in previous studies. 8.

Previous experiments have indicated that the suppression of functionality after exercise is often accompanied by the decrease in CD19^+^ levels 35 and that acute exercise results in the mobilization of immature CD19^+^ (naïve B cells), with no change in plasma cell counts before and after exercise 36. This might explain why the CD19^+^ levels did not change immediately postexercise but significantly decreased at Post-2 for both groups in the present study. In addition, this study revealed that for all participants, the CD56^+^ counts were significantly higher at Post-0 than at baseline, and the counts rapidly decreased at Post-2. The present findings provide strong support for the open window theory of immune responses and are consistent with those of other studies 6,37. Furthermore, the results reveal that for all participants, both the CD4^+^ and the CD8^+^ levels significantly decreased at Post-2, whereas immediately after exercise, only the CD8^+^ levels significantly increased. A previous study indicated that the CD4^+^/CD8^+^ ratio is an indicator of immune competence in individuals with infection 38. A longitudinal study confirmed that a CD4^+^/CD8^+^ ratio of ≤1.0 indicates low immune function in patients with infection 39. Our study revealed that the CD4+/CD8+ ratio for all participants significantly decreased immediately after exercise, but the ratio returned to that at baseline at Post-2. A previous study in our laboratory indicated that in participants performing strenuous endurance exercise (65% V̇O_2max_ for 2 hours) on a cycling ergometer, the CD4^+^/CD8^+^ ratio was significantly lower at all time points within 24 hours postexercise, with the exception of a brief increase at 2 hours postexercise, than at baseline 25. The current results indicate that maca extract supplementation did not effectively enhance immune function following EEE. However, the exercise intervention facilitated rapid recovery of immune responses following the open window phenomenon and mitigated potential long-term immune suppression.

The significant decrease in CD4^+^ levels noted in the present study for all participants at Post-2 aligns with previous results regarding acute changes in T lymphocyte counts after exercise 3. Although maca supplementation did not increase CD4^+^ counts as expected, IFN-γ secretion from T lymphocytes increased in both groups at Post-0, and the maca group exhibited nearly significantly higher levels than the placebo group did. Additionally, the IFN-γ levels peaked immediately after exercise and declined within 1 hour postexercise. This finding aligns with those of studies implementing shorter exercise durations 5,40 that have reported an upward trend. The finding contrasts with the results of studies implementing longer exercise durations 29,41, which have reported IFN-γ to significantly decrease within 24 hours. Therefore, the exercise duration may be a key factor influencing IFN-γ levels. Moreover, the finding of higher IFN-γ levels in the maca group aligns with that of an earlier rat study that revealed that maca extract increased IFN-γ levels after ovariectomy 42. Polysaccharides and alkaloids in maca promote CD4^+^ T-cell proliferation and increase IFN-γ and IL-2 secretion, improving the Th1/Th2 balance, even under suppressed immune conditions 19,43. IFN-γ regulates immune function by promoting CD56^+^ activity, increasing antigen presentation, activating inducible nitric oxide synthase (iNOS), and promoting Th1-cell differentiation through T-bet upregulation 4. Prolonged exhaustive exercise lowers type-1 T cell counts and IFN-γ levels while increasing IL-4 production, elevating the risk of upper respiratory symptoms 44. Therefore, the increase in IFN-γ levels in the maca group at Post-0 in this study may positively affect immune function.

This study revealed an inverse trend for IL-4 and IFN-γ levels at different time points following EEE, which may be attributable to the inhibition of Th1 cells by IL-4 and the subsequent suppression of IFN-γ production 3. In addition, the results of this study indicated that following EEE, blood IL-2 levels significantly decreased at Post-0 and Post-2 compared with baseline levels. This finding is in line with that of Weinstock *et al.* (1997), who reported a significant decrease in IL-2 levels at 1 hour after strenuous exercise in trained men 45. Furthermore, the results of this study indicated that TNF-α, IL-6, IL-8, and IL-10 levels did not significantly increase following EEE, which contrasts with previous findings 12. The discrepancy may stem from the use of PBMCs instead of plasma or serum in this study. Early increases in serum IL-6 levels after intense exercise are primarily driven by IL-6 secretion from damaged muscle fibers, not PBMCs 46,47. In the current study, TNF-α levels slightly decreased below baseline levels at Post-2, and the levels exhibited a delayed increase at Post-24. Moreover, a delayed increase was noted in IL-6 at 2 hours postexercise. IL-8 and IL-10 levels significantly increased at Post-2 in both the maca and the placebo groups; this finding is consistent with the meta-analysis results but differs from those of other studies 12,18. This discrepancy may be due to our participants having different inflammatory conditions than those of other studies, which were induced by the lipopolysaccharide (LPS) endotoxin. A rapid increase in blood IL-6 levels occurs after intense exercise that is attributable to its direct secretion by damaged muscle fibers rather than by PBMCs. Therefore, maca extract might not achieve an anti-inflammatory effect by inhibiting the secretion of cytokines by PBMC. This study is the first to investigate the changes in the cytokines secreted by PBMCs at multiple time points within 24 hours after exercise in patients with 12-week maca extract supplementation. To explore the anti-inflammatory effects of maca extract, future research should analyze the role of the NF-κB pathway in macrophages and T cell-dominated inflammatory responses by examining protein phosphorylation status and using targeted culture stimuli such as LPS or Concanavalin A.

Apart from various strengths, our study also includes some limitations. First, the small sample size of this investigation limits the generalizability of our findings. Although the matched-pair design ensured equivalence in baseline aerobic capacity and fitness levels between the maca and placebo groups, a larger sample size is needed to confirm these results and further validate the observed effects of maca supplementation on immune and cytokine responses. Second, the exclusion of female participants restricts the applicability of our findings to a broader population. This decision was made to minimize variability caused by hormonal fluctuations, which could influence immune and inflammatory responses to exercise. Future studies should include female participants to provide a more comprehensive understanding of maca's effects across genders. These limitations have been acknowledged to encourage further research and provide a balanced interpretation of the study's findings.

## Conclusions

Our results indicate that a 60-minute EEE on a treadmill effectively induced the open window phenomenon, characterized by transient immune suppression in healthy men. Continual supplementation with 2.25 g of maca extract twice daily for 12 weeks exerted a positive effect by enhancing IFN-γ levels secreted by PBMCs immediately postexercise, highlighting its potential for immune modulation. However, no additional benefits were observed in terms of reducing the inflammatory response or improving other immune-related indicators resulting from EEE.

Based on these findings, maca supplementation may be a useful strategy for athletes seeking to enhance immune function, particularly during periods of intense training or competition. We recommend a dosage of 2.25 g of maca extract taken twice daily, ideally incorporated into a balanced diet, for a minimum of 12 weeks to achieve measurable benefits in IFN-γ secretion. Target populations may include endurance athletes or individuals with compromised immune function following prolonged exercise. Future studies should explore the effects of maca supplementation in diverse populations, including females, while incorporating comprehensive immune and performance metrics, such as long-term inflammatory markers and exercise economy. To further build upon these findings, investigating the regulatory pathways of maca's bioactive compounds, particularly polysaccharides and alkaloids, in modulating exercise-induced immune responses and cytokine activity is also essential. These efforts would clarify maca's mechanisms of action and expand its applications in promoting immune function and athletic performance.

## Figures and Tables

**Table 1:** Participant characteristics.

Variable	Maca Group	Placebo Group	*p* value
Age (years)	23.89 ± 1.62	25.09 ± 4.21	0.398
Body height (cm)	174.16 ± 5.04	172.85 ± 6.30	0.620
Body weight (kg)	69.09 ± 10.75	66.83 ± 12.11	0.668
First V̇O_2max_ (mL/kg/min)	51.78 ± 4.76	54.00 ± 5.12	0.332
Second V̇O_2max_ (mL/kg/min)	48.67 ± 5.32	49.36 ± 4.95	0.765
70% V̇O_2max_ speed (km/h)	9.29 ± 1.57	9.45 ± 1.29	0.809

Data are presented as means ± standard deviations (SDs); Maca group: n = 9. Placebo group: n = 11.
